# A clinical case study of seven patients of autonomic dysfunction in post COVID-19 conditions with fever as the main clinical symptom: a case series

**DOI:** 10.1080/07853890.2024.2402943

**Published:** 2024-12-03

**Authors:** Liu Haihong, Xu Nannan, Meng Xiangzhu, Wang Gang

**Affiliations:** a Department of Infectious Disease, Qilu Hospital of Shandong University, Jinan, Shandong, China; b Department of Gastroenterology, Shandong Rongjun General Hospital, Jinan, China

**Keywords:** Fever, autonomic dysfunction, post COVID-19 condition

## Abstract

**Background:**

Many publications have reported that acute COVID-19 infection can cause autonomic dysfunction. In this series, we described seven patients who had recurrent fever after acute COVID-19 infection, and the possible pathophysiological basis is autonomic dysfunction.

**Patients:**

This was a retrospective study conducted at the Qilu Hospital of Shandong University from January 2023 to March 2023. Patients who were hospitalized in the Department of Infectious Diseases with a diagnosis of fever of unknown origin.

**Results:**

Between January and March 2023, a total of seven patients with autonomic dysfunction in post-COVID condition, who had recurrent fever accompanied by electrolyte imbalances and other manifestations of autonomic dysfunction. The median age of these patients was relatively high, and they were mostly indoor workers with comorbidities such as diabetes and chronic hypertension. Physical cooling and correction of electrolyte imbalances with medication were effective treatments.

**Conclusions:**

The COVID-19 infection can lead to autonomic dysfunction, which manifests not only as tachycardia and blood pressure abnormalities, but may also be the pathophysiological mechanism underlying recurrent fever in post-COVID cases.

## KEY MESSAGES

- In this series, we describe seven patients who had recurrent fever after acute COVID-19 infection, and the possible pathophysiological basis is autonomic dysfunction.
- This case series highlights the concept of long COVID syndrome and provides a basis for clinicians to identify and treat these patients appropriately.

## Background

The coronavirus has severely impacted the global economy and disrupted daily life over the past three years. Many people who contract the virus experience acute flu-like symptoms and recover within a short period of time. However, some individuals may develop persistent symptoms or experience a relapse after a period of recovery, lasting for four weeks or even several months, this condition called post-COVID-19 condition (PASC) [1–3]. This can occur in any patient with a COVID-19 infection, regardless of the severity of their symptoms or whether they were asymptomatic. Common symptoms of PASC include fatigue, difficulty breathing, tachycardia, orthostatic hypotension, and memory impairment. The clinical manifestations of post-COVID-19 are variable and often involve multiple organs. While the underlying pathophysiological mechanisms of PASC are currently unclear, literature suggests that dysfunction of the autonomic nervous system may be a potential cause [4].

Several studies have described COVID-19 damage to the autonomic nervous system, which can result in symptoms such as orthostatic hypotension, tachycardia, fatigue, difficulty breathing, and cold and sweaty extremities [5–7]. While autonomic dysfunction is common in post-COVID-19 patients, reports of recurrent fever are scarce. In this series, we describe seven patients from a single center in China who presented with persistent or recurrent fever following acute COVID-19 infection, despite thorough investigations revealing no other explanation for their fever. Notably, all of these patients exhibited clear signs of autonomic dysfunction(AD). Our aim was to raise awareness among clinicians for this subset of patients and encourage appropriate treatment.

## Case presentation

### Methods

This was a retrospective study conducted at the Qilu Hospital of Shandong University from January 2023 to March 2023. Patients who were hospitalized in the Department of Infectious Diseases with a diagnosis of fever of unknown origin. This study was approved by the ethics committee of Qilu Hospital of Shandong University. The study was performance in accordance with the Declaration of Helsinki and all methods were performed in accordance with the relevant guidelines and regulations. Each subject read and subscribed a written informed consent for treatment, collection and use of data or samples, and being included in scientific publications. Information that might identify a patient is not disclosed in the article. The ethics review approval number is: KYLL-202111-168.

### Study population

All patients admitted met the criteria for fever of unknown origin (FUO) and post-COVID conditions. Patients who were COVID-19 positive, had no prior history of COVID-19, had a fever duration of less than 4 weeks, or had incomplete medical records were excluded from the analysis. After diagnostic evaluations, patients with fever caused by AD were included in the analysis.

### Standard of diagnosis

#### Fever of unknown origin (FUO)

In 1961, Petersdorf and Beeson defined FUO as a temperature of 38.3 °C or higher for at least 3 weeks without a diagnosis, despite 1 week of inpatient investigations. With the evolution of health care delivery in the ambulatory setting, Durack and Street’s revised criteria shortened the investigation period to 3 inpatient days or at least 3 outpatient visits [8].

#### Post-COVID-19 condition

Post-COVID-19 condition occurs in individuals with a history of probable or confirmed SARS-CoV-2 infection, with symptoms that last for at least 4 weeks and cannot be explained by an alternative diagnosis [2].

### Confirmation of COVID-19 infection status

These patients underwent COVID-19 nucleic acid and antibody testing to confirm their COVID-19 infection status. Negative COVID-19 nucleic acid and positive IgG antibody rule out the status of COVID-19 infection and confirm that the patient has been infected with COVID-19.

### Collection of relevant data

Once the cases were identified, all available clinical data were reviewed to collect demographic and baseline characteristics (age, gender, occupation, medical history, and COVID-19 vaccination history), details of acute COVID-19 infection (time, symptoms, treatment, outcomes and diagnostic methods), and clinical features relevant to the current fever episode (duration of fever, interval from acute COVID-19, main symptoms, especially those related to autonomic dysfunction, and relevant laboratory investigations).

### Autonomic testing

Following the previous method, autonomic nervous function testing was performed from three aspects [9]: sudomotor the thermoregulatory sweat test (TST), the sympathetic skin responses (SSR), and cardiovascular reflex test (heart rate response to standing up and blood pressure response to standing up). Due to limitations in experimental conditions, the tilt test was replaced by the standing test.

The TST is a sensitive qualitative test of sudomotor function by quantifying areas of anhidrosis across the entire anterior body surface. The commonly used iodine starch test involves mixing 1.5 g of iodine, 10 ml of castor oil, and 100 ml of 95% alcohol to create a dilute iodine tincture, which is applied to the skin and allowed to dry before dusting with starch. The core body temperature is raised to 38 °C in a standardized environment, and the sweating range is evaluated by observing the blue color of the starch after the skin becomes sweaty.

SSR is one of the electrophysiological methods used to test the autonomic nervous system function, which refers to the skin reflex potential that appears after the body receives stimuli. It originates from the impulse released by sympathetic nerve fibers, which induces synchronous activity of sweat glands.

The cardiovascular reflex test includes changes in heart rate and blood pressure during postural changes, which evaluate the cardiovagal function and cardiovascular adrenergic function, respectively. After resting in a supine position for 20 min, the subject stands up and the longest RR interval around the 30th heartbeat and the shortest RR interval around the 15th heartbeat are measured. The ratio of these two values is defined as the 30/15 ratio, and a value less than 1 is considered positive. The subject is then instructed to stand for one minute, and a drop in systolic blood pressure of 20 mmHg or a drop in diastolic blood pressure of 10 mmHg or more is considered orthostatic hypotension.

### Telephone follow-up

After discharge from the hospital, we conducted a telephone follow-up with patients with AD fever 4 weeks later to inquire about their symptom improvement and the results of their follow-up exams.

### Statistic analysis

In addition to collecting case data, we also conducted simple statistical analysis. Descriptive statistics were reported for continuous variables using the median (interquartile range), and for categorical variables using counts (percentages).

## Results

Between January 2023 and March 2023, a total of 40 patients with fever of unknown origin for more than 4 weeks after acute COVID-19 infection were admitted. We followed the diagnostic and treatment guidelines for FUO published in the English medical journal in 2021. Firstly, we conducted initial evaluations on patients, including detailed medical histories and physical examinations, to confirm the fever situation and provide supportive treatment. We also conducted basic laboratory tests such as complete blood count, erythrocyte sedimentation rate (ESR), and C-reactive protein (CRP), a complete metabolic workup, blood cultures (2 sets), as well as HIV testing, echocardiography and CT scans of the chest, abdomen, pelvis and brain. Based on the symptoms and examination results, we further evaluated the patients for fungal, tuberculosis, rheumatological and brucellosis-related infections.

After the diagnosis and treatment of fever of unknown origin in all patients, 21 patients were diagnosed with infective endocarditis, brucellosis, lymphoma, and other conditions. In addition, 19 patients were not able to identify the cause of the fever. However, after evaluating the basic information, clinical manifestations, and laboratory tests of the patients, we found a group of seven patients with similar clinical characteristics. Ultimately, we considered it as fever caused by autonomic dysfunction after COVID-19. The demographic and clinical characteristics of these seven patients are presented in Table 1.

**Table 1.** Baseline characteristics and clinical presentations of fever patients with autonomic nervous system dysfunction.

Variables		Patient 1	Patient 2	Patient 3	Patient 4	Patient 5	Patient 6	Patient 7
Population classification		Retired	Retired	Retired	Retired	Retired	Retired	Retired
Age		62	64	91	74	75	85	78
Gender		Male	Male	Male	Male	Male	Female	Male
Vaccination status		No	Yes	No	No	Yes	No	Yes
Interval between COVID-19 infection and recurrence of fever		15	32	19	12	18	0	27
Underlying chronic diseases		CHD,DM, cerebral infarction	DM	HBP	HBP	HBP, DM, CHD	HBP, cerebral infarction,	HBP, DM
Clinical status of the acute phase of COVID-19		mild case: fever, respiratory symptom	ordinary type: fever, respiratory symptom, pulmonary infection	mild case: fever, gastrointestinal symptom, lacking in strength	mild case: fever, gastrointestinal symptom, dysgeusia	ordinary type: fever, respiratory symptom, pulmonary infection	ordinary type: fever, respiratory symptom, gastrointestinal symptom, pulmonary infection	asymptomatic infection
Main symptoms after COVID-19		Fever,frequency of urinatior, lacking in strength	Fever, Poor sleep, hypohidrosis	Fever, lacking in strength, hypohidrosis	Fever, Increased urination at night	Fever, swirl	Fever, cough	Fever
AD related symptoms	postural hypotension	Yes	Yes	No	Yes	No	No	No
	polycardia	Yes	No	No	No	No	Yes	No
	gastrointestinal symptom	No	No	Yes	No	Yes	No	No
	Bladder symptom dysuresia^a^	No	Yes	Yes	No	Yes	No	Yes
	hypohidrosis	Yes	Yes	Yes	Yes	Yes	Yes	Yes

^a^Bladder symptom dysuresia include frequent micturition,Urine retention and uroclepsia.

CHD: coronary heart disease; HBP: high blood pressure; DM: diabetes mellitus.

### Manifestations of acute COVID-19 infection

Three patients developed mild acute symptoms of SARS-CoV-2 infection and None of the three patients was hospitalized. In addition to symptoms of fever, one patient had significant pharyngeal pain, the other two had gastrointestinal symptoms, and one patient had taste disturbance. All patients were treated with oral antipyretic drugs and Chinese patent medicine. Their body temperature returned to normal in 3–5 days, and other symptoms disappeared within 1 week. One patient had no fever or upper respiratory symptoms. Antibody testing confirmed that this patient had a previous infection, and the time of infection was estimated based on information provided by the patient’s family. The other three patients were complicated with lung infection during the acute period of COVID-19, all of whom had obvious cough. All of them were hospitalized. After anti-inflammatory and symptomatic supportive treatment, the body temperature of all patients recovered. After discharge, chest CT was reexamined and the lung inflammation disappeared. Prior to SARS-CoV-2 infection, three patients had been fully vaccinated, while four patients had not received any vaccines.

### Manifestations of post-COVID-19

One patient had persistent fever after acute COVID-19 infection, while the rest of the patients had a period of time when their body temperature returned to normal before experiencing recurrent fever. Two patients had no discomfort and were in normal condition after acute COVID-19 infection. The remaining four patients did not experience recurrent fever for a period of time after acute COVID-19 infection, but experienced discomfort such as fatigue, dizziness, sleep disturbances, increased urination and gastrointestinal symptoms. Most of the patients were complicated with the clinical manifestations of autonomic dysfunction. All the patients were hospitalized in local hospitals for diagnosis and treatment due to fever. No definite diagnosis was made after the improvement of imaging, rheumatism, infection and other related laboratory tests, but they were given anti-infection treatment and still had repeated fever.

### Autonomic function testing

Details of autonomic function testing are summarized in Table 2. TST and SSR was abnormal in 7/7(100%), cardiovagal testing was abnormal in 2/7 (42.9%), and cardiovascular adrenergic function was abnormal in 3/7 (42.9%) patients.

**Table 2.** The details of autonomic function testing.

Patients	Sudomotor		Cardiovagal	Adrenergic
	TST	SSR	Heart rate response to standing up	Blood pressure response to the standing up
1	+	+	+	+
2	+	+	–	+
3	+	+	–	–
4	+	+	–	+
5	+	+	–	–
6	+	+	+	–
7	+	+	–	–

### Fever patients with autonomic dysfunction

These patients were generally elderly, with a median age of 75 years (range 62–91 years), and all were retired. They lived in environments with air conditioning and heating, which kept them warm in winter and cool in summer. Laboratory results showed that their white blood cell counts were not elevated and inflammatory markers such as CRP and ESR were not significantly abnormal. All patients had electrolyte imbalances, with hyponatremia observed in all cases, with a median sodium concentration of 120 mmol/L (range 111–126 mmol/L). Some patients also had hypokalemia (71.4%). Most patients exhibited symptoms of autonomic dysfunction, including orthostatic hypotension (57.1%), tachycardia (42.9%), anhidrosis (100%), gastrointestinal symptoms (28.6%), and bladder dysfunction (57.1%). Physical cooling with ice packs was effective in reducing their body temperature.

Seven patients experienced a recurrence of fever after resolution of acute symptoms of SARS-CoV-2 infection, with a median interval of 18 days (range 0–32 days). We reviewed the medical history of all patients and found that most of them had a history of hypertension, diabetes and cerebral infarction. None of the patients showed any signs of autonomic nervous system dysfunction or related diseases.

### Therapy

**Figure 1.**
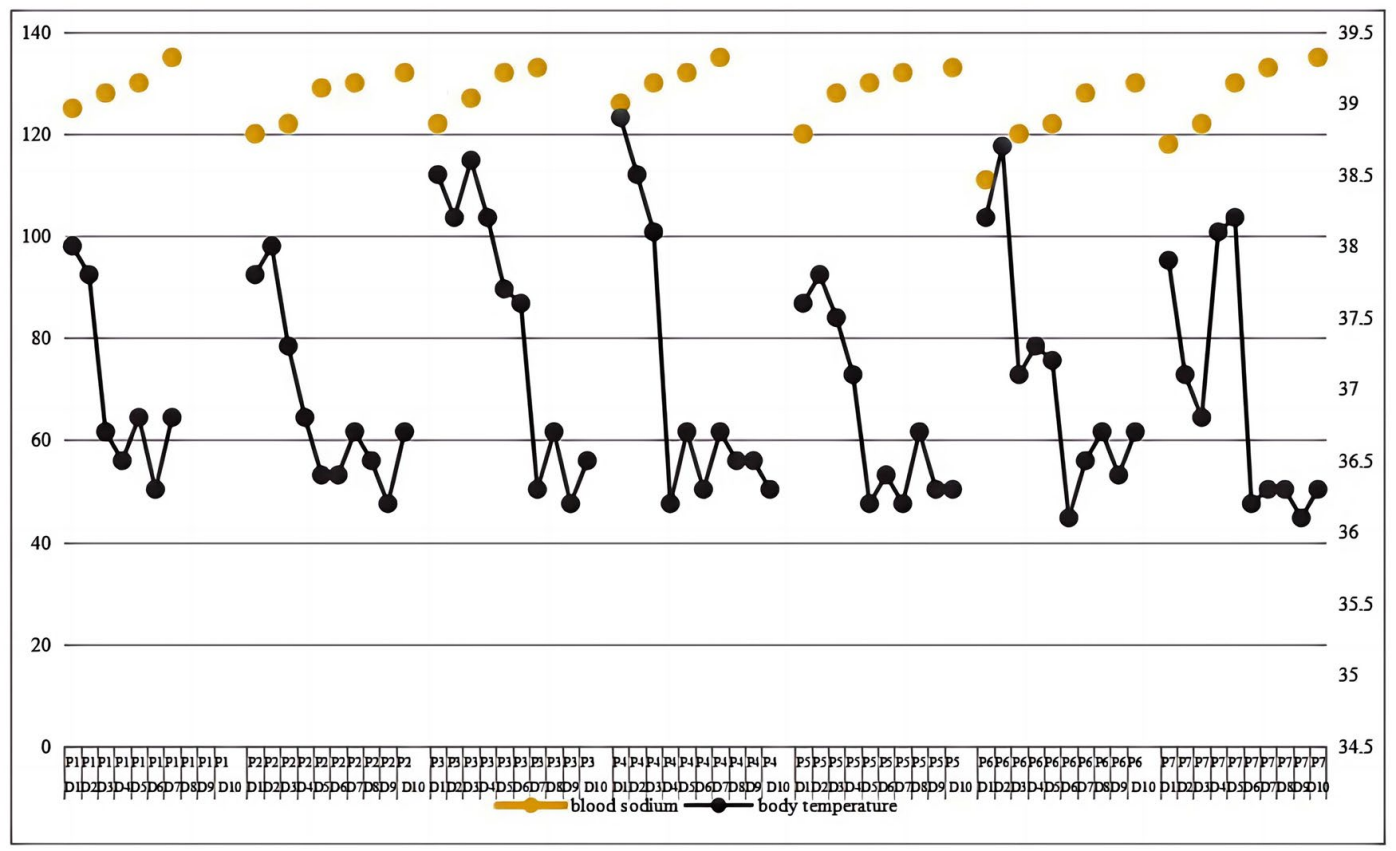
Changes in body temperature and serum sodium levels during hospitalization. On the top: Changes of blood sodium in patients after treatment with concentrated sodium supplementation (yellow polka-dot). The gray line at the bottom is the trend chart of the patient’s temperature. As can be seen from the figure, the change of blood sodium is correlated with the change of body temperature. With the improvement of body temperature, blood sodium also returns to normal.

All patients received physical cooling therapy using ice blankets and electrolyte replacement therapy with sodium and potassium supplements. All patients’ body temperature returned to normal range after treatment, with recovery time ranging from 1 to 6 days, with a median time of 3 days. Three of the patients stopped using the ice blanket for physical cooling before discharge, and their body temperatures were maintained within the normal range, while the other four patients needed to continue using the ice blanket for physical cooling to maintain their normal body temperatures. After receiving concentrated sodium therapy, the patients’ blood sodium levels could be maintained above 130 mmol. In all patients, blood sodium levels rose as body temperature returned. Body temperature and blood sodium levels are shown in Figure 1. From Figure 1, we can see that sodium recovery is accompanied by body temperature recovery. Three patients with postural hypotension had mild symptoms and were treated with non-pharmacological methods, such as increasing water intake, eating smaller meals more frequently, increasing salt intake, and appropriate physical exercise.

### Telephone follow-up

A month after the patient left the hospital, we conducted telephone follow-up on these 7 patients, among whom 5 patients recovered and did not show any signs of fever and hyponatremia after discharge. Another 91-year-old male patient needed physical cooling with ice blankets to maintain a normal body temperature. The remaining patient was readmitted to the local hospital due to recurrence of fever, and the patient’s family provided the following hospitalization information. There was no evidence of infection this time. The patient still had hyponatremia and hypokalemia. Anti-infection treatment was ineffective, while physical cooling was effective.

## Discussion and conclusion

Seven patients have been experiencing recurrent fever after recovering from COVID-19. We conducted relevant tests and examinations to identify the possible cause and mechanisms behind this phenomenon, taking reference from detailed investigations of fever of unknown origin [10]. Blood cultures were negative, and inflammatory markers were not elevated, while CT scans did not reveal any signs of inflammation, ruling out bacterial infections. Comprehensive viral testing (including hepatitis B and C, HIV, EBV and CMV) did not show any abnormalities. Autoimmune diseases were also ruled out as levels of anti-nuclear antibodies, rheumatoid factor and anti-cyclic citrullinated peptide antibodies were not elevated. Complement levels were not increased. With no elevated tumor markers and no evidence of tumors found in chest, abdomen and pelvic CT scans, we concluded that the patients’ long-term intermittent fever was a post-COVID-19 sequelae, based on their clinical characteristics.

There were few reports of patients experiencing recurrent fever after COVID-19 [11], but there are also numerous reviews analyzing possible mechanisms for post-COVID-19 sequelae, with many hypotheses explaining the persistent symptoms: inflammation, autoimmune reactions and delayed clearance of the virus [2]. In clinical practice, we have found that some patients with post-COVID-19 fever have elevated inflammation markers, and we attribute this to abnormal inflammation caused by COVID-19. However, the cases we are reporting on here are different: these patients do not have any obvious abnormalities in inflammation markers or organ function, nor do they show any signs of immune system dysfunction. The clinical manifestations that accompany fever are electrolyte imbalances, mainly low sodium. Additionally, these patients also exhibit some degree of autonomic dysfunction, such as sleep disorders, bowel abnormalities and orthostatic hypotension. Therefore, we conducted further tests related to autonomic function and found that all patients had abnormalities in SSR and TST tests. The impact of COVID-19 on the autonomic nervous system has also been reported in some literature, including postural tachycardia syndrome (POTS) [12–14], small fiber neuropathy with orthostatic cerebral hypoperfusion syndrome [15], paroxysmal hypothermia and hyperhidrosis [16]. A prospective study by Alex Buoite Stella and others pointed out that the most significant manifestation of autonomic dysfunction after COVID-19 was intolerance to standing and sweat dysfunction [7]. Temperature regulation is an important response of the autonomic nervous system to cold and heat stress. Autonomic dysfunction can cause widespread anhidrosis, leading to impaired heat release capacity. Therefore, we propose a hypothesis that COVID-19 virus may cause autonomic nervous system damage, resulting in sweating dysfunction and recurrent fever in patients. We proved that these patients had sweating disorder by TST experiment, and abnormal skin sympathetic reflex by SSR experiment.

Some patients in this group also exhibited a notable feature of low sodium, and we performed a diagnostic evaluation according to the 2014 ESE/ESICM/ERBP Clinical Practice Guidelines for the Diagnosis and Management of Hyponatremia [17]. We conducted tests for blood and urine osmolality, cortisol, 24-hour urine sodium and ultimately confirmed the presence of syndrome of inappropriate antidiuretic hormone secretion (SIADH) in these patients. Adrenocorticotropic hormone (ACTH) secretion was normal, ruling out ADH secretion abnormalities caused by hypothalamic-pituitary dysfunction. In addition to hypothalamic-pituitary dysfunction, various diseases can cause SIADH, such as malignant tumors, pulmonary diseases, and neurological diseases. We hypothesized that the dysfunction of the autonomic nervous system input pathway in these patients could affect ADH secretion and cause hyponatremia. Kloesel previously reported a case of Guillain-Barré syndrome (GBS) in which severe hyponatremia was an early symptom and proposed a hypothesis that GBS-induced autonomic nervous system dysfunction could lead to water and sodium balance disturbances. Some literature suggests that autonomic dysfunction may cause conduction blockage of the sinoatrial stretch receptors, which may lead to inappropriate secretion of antidiuretic hormone and renin and may cause electrolyte imbalances [18].

Diseases with autonomic dysfunction include primary causes such as Parkinson’s disease, multiple system atrophy, Lewy body dementia, pure autonomic failure and secondary causes such as diabetes, amyloidosis and immune-mediated diseases [19]. These patients need to be distinguished from diseases that cause autonomic dysfunction. These patients do not have peripheral sensory or motor disorders, only widespread autonomic dysfunction (orthostatic hypotension, sweating disorders, bladder dysfunction etc.), without other neurological signs. After comprehensive evaluation, Parkinson’s disease, multi-system obscene disorder, Lewy body dementia and other diseases were excluded, and we considered it to be pure autonomic failure.

Our group of patients has common characteristics, and based on the viewpoints of other researchers, we proposed a hypothesis that post-COVID-19 recurrent fever is caused by autonomic dysfunction resulting from acute COVID-19 infection, which was confirmed clinically. This study has several limitations. First of all, we need to collect more patients with similar conditions and conduct further evaluations, such as skin nerve biopsies and ambulatory blood pressure monitoring, to support our hypothesis. Evidence of decreased or absent intraepidermal nerve fibers in percutaneous skin biopsies is a minimally invasive technique that can be used to support the diagnosis of small fiber neuropathy. The sensitivity and specificity of skin biopsy for the diagnosis of small fiber neuropathy were 78–92% and 65–90%, respectively [20]. Ambulatory blood pressure monitoring provides some advantages over the measurement of orthostatic vital signs in the autonomic laboratory or during office visits. It allows for ‘real-life’ assessment while patients perform daily activities,and also for follow-up [21]. High variability of blood pressure with low heart rate variability over a 24 h may allow for accurate identification of patients with autonomic neuropathy with autonomic failure [22].

In addition, we need to further evaluate whether the patient has only peripheral autonomic dysfunction or a combination of central autonomic dysfunction. We found that some patients completely returned to normal body temperature after treatment, while some patients still needed ice blanket to maintain physical cooling. Is this the continuous effect of the novel coronavirus? We are not sure what the real mechanism is. Larger cohort studies, different patient groups or deeper physiological studies are needed.

The treatment of autonomic neuropathies constitutes a comprehensive system that encompasses disease-modifying therapies, non-pharmacological interventions, and targeted pharmacological treatments [22]. Non-pharmacological Management primarily involves adjustments to one’s lifestyle. Encouraging patients to engage in moderate physical activity, avoiding overexertion, and maintaining good sleep habits are all essential for improving autonomic function. For patients with orthostatic hypotension, in addition to wearing long compression stockings, slow and steady movements when changing positions are essential. Adequate hydration and salt intake are also crucial.Pharmacotherapy involves administering medications that are targeted at alleviating specific symptoms or conditions.Such as midodrine (alpha-agonists): This drug is mainly used to increase peripheral vascular resistance and increase blood pressure by contracting blood vessels. Beta-adrenergic receptor blockers: This type of drug is suitable for patients with tachycardia because they can effectively reduce heart rate and reduce the burden on the heart.In addition patients with autonomic neuropathy often experience emotional issues such as anxiety and depression. Therefore, providing psychological support and necessary counseling is crucial for their overall recovery. In summary, the treatment of autonomic neuropathy necessitates a holistic approach that considers multiple factors and employs individualized, comprehensive treatment plans. Through scientific disease management, reasonable non-pharmacological interventions, and precise pharmacological treatments, we can significantly improve patients’ quality of life and alleviate their disease burden.

There are many patients with fever of unknown origin after acute COVID-19 and there are many reasons. This case report series provides a basis for clinicians to identify and treat these patients appropriately.

## Data Availability

All data of the seven cases described in the manuscript are present in the paper.
